# Platinum-based neoadjuvant chemotherapy in BRCA1-positive breast cancer: a retrospective cohort analysis and literature review

**DOI:** 10.1186/s13053-018-0092-2

**Published:** 2018-04-27

**Authors:** Nikolai Havn Sæther, Elina Skuja, Arvids Irmejs, Jelena Maksimenko, Edvins Miklasevics, Gunta Purkalne, Janis Gardovskis

**Affiliations:** 1 Riga Stradins University, Riga, Latvia; 2 Pauls Stradins Clinical University Hospital, Clinic of Oncology, Riga, Latvia; 3 Pauls Stradins Clinical University Hospital, Breast unit, Riga, Latvia

**Keywords:** Neoadjuvant, Cisplatin, Platinum, BRCA1, Breast cancer, pCR

## Abstract

**Background:**

There is increasing evidence of high platinum sensitivity in *BRCA*-associated breast cancer. However, evidence from randomized trials is lacking. The aim of this study was to analyze the results of platinum-based chemotherapy for BRCA1-positive breast cancer in a neoadjuvant setting.

**Methods:**

A retrospective study was performed by obtaining information from patient files. The results were compared with the available data from a literature review.

**Results:**

Twelve female patients with *BRCA1* gene mutations who had stage I to III breast cancers were eligible for evaluation. They received platinum-based neoadjuvant chemotherapy between 2011 and 2016. Eleven patients received a combination of cisplatin and doxorubicin, and one patient received carboplatin and docetaxel. All patients underwent mastectomy after chemotherapy. Ten patients (83%) achieved pathological complete remission (pCR). The observed pCR rate was comparable to existing results found in similar studies.

**Conclusion:**

The results of the study confirm the high pCR rate in BRCA1-positive breast cancer after platinum-based neoadjuvant chemotherapy. Larger randomized studies and longer follow-up times are necessary to evaluate the role of platinum-based therapies in BRCA1-positive breast cancer.

## Background

Hereditary breast cancer accounts for approximately 5–10% of all breast cancer cases. *BRCA1/2* gene mutations are responsible for approximately two-thirds of hereditary breast cancers [1]. The *BRCA* genes are tumor suppressor genes that encode proteins that are involved in homologous recombination (HR) repair. HR is the most common repair mechanism for double strand breaks during the DNA replication process. Mutations in the *BRCA* gene result in genomic instability and subsequent malignant processes. The platinum agent introduces intra-strand crosslinks. Due to faulty DNA-repair proteins from the pathogenic *BRCA1* mutation, cellular protein synthesis and replication are reduced, and eventually, cells undergo apoptosis. BRCA-associated breast cancers are often triple negative breast cancers (TNBCs). Due to significant differences in chemotherapy sensitivity that depend on hormone receptor status, various chemotherapy regimens have been tested in adjuvant and neoadjuvant settings for BRCA-associated breast cancer. Attempts to understand the role of *BRCA* genes in and sensitivity of cancer cells to DNA damaging agents in *BRCA* mutation carriers have reestablished interest in platinum agents in the context of breast cancer treatment.

Several studies have shown the sensitivity of *BRCA*-related breast cancer to platinum agents, such as cisplatin and carboplatin, and more studies will follow. The ESMO clinical practice guidelines [2] for primary breast cancer published in 2015 do not recommend platinum agents for routine use for BRCA1/2 mutation carriers; however, these guidelines state that adding a platinum compound to NACT, after discussion with the patient, is acceptable. The ESO-ESMO 3rd international consensus guidelines for advanced breast cancer (ABC3) [3] published on December 5th, 2016, recommend a platinum regimen chemotherapy for *BRCA*-associated TNBC if not previously administered.

The aim of this study was to retrospectively evaluate platinum-based neoadjuvant chemotherapy for BRCA1-positive breast cancers.

## Methods

A retrospective evaluation was made of platinum-based neoadjuvant chemotherapy in breast cancer patients with *BRCA1* gene mutations who were treated at the breast unit at Pauls Stradins Clinical University Hospital (PSCUH). Patient data were collected from the hospital database, along with the respective patient history and chemotherapy chart from the Clinic of Oncology at PSCUH. This retrospective evaluation was approved by Riga Stradins University Ethical committee (Nr.55/ 27.10.2016). Patients with diagnosed invasive breast cancers and confirmed *BRCA1* gene mutations who received platinum-based chemotherapy in the neoadjuvant setting were included in the evaluation.

Genomic DNA was isolated from peripheral blood cells using the FlexiGene DNA Kit (Qiagen, Germany). Screening of the three most common *BRCA1* mutations in Latvia, namely, c.4035delA (BIC: 4154delA), c.5266dupC (BIC: 5382insC) and c.181 T > G (BIC: 300 T > G/C61G), was performed by multiplex PCR.

Insufficient patient data was the exclusion criterion. Pathological complete response was defined as the complete disappearance of an invasive tumor. The follow-up time was the time from mastectomy to February 2017.

### Study population

Twelve BRCA-associated patients were eligible for this evaluation during the period from 2011 to February 2017.

The clinical characteristics of the patients are presented in Table 1. All patients except one received a regimen of 50–75 mg/m^2^ cisplatin and 50–60 mg/m^2^ doxorubicin (one patient received carboplatin AUC5 and 75 mg/m^2^ docetaxel) every 3weeks for three to 6 cycles. The treatment regimens are presented in Table 2. Four patients had received postoperative radiation therapy. One patient had received additional adjuvant cisplatin and doxorubicin for 2 cycles. Seven patients (58%) received 6 cycles with platinum agents in all cycles.

**Table 1:** Basal clinical characteristics of neoadjuvant patients, *n* = 12

Characteristic	No.	Percent
Age, years		
Mean	38	
Range	28–56	
BRCA1 mutation		
Exon 20 c.5266dupC (BIC: 5382insC)	8	66
Exon 11 c.4035delA (BIC: 4154delA)	4	34
Clinical tumor size		
T1	5	42
T2	5	42
T3	2	17
Clinical nodal status		
N0	6	50
N1	5	42
N2	0	0
N3	1	8
Estrogen receptor level		
Positive	1	8
Negative	10	83
Missing	1	8
Progesterone receptor level		
Positive	1	8
Negative	10	83
Missing	1	8
HER2 status		
Positive	1	8
Negative	8	66
Missing	3	25
Differentiation		
Grade I	0	0
Grade II	4	33
Grade III	7	58
Missing	1	8
Histology		
Ductal	10	83
Medullary	1	8
Missing	1	8
Chemotherapy		
Cisplatin + Doxorubicin	11	92
Carboplatin + Docetaxel	1	8
Pathological remission		
Complete	10	83
Partial	2	17
Salpingoopherectomy		
No	7	58
Yes	5	42
Postoperative radiation		
No	8	66
Yes	4	33

**Table 2:** NACT regimens of patients evaluated

Agents	Dosages	Cycles	No. of patients
Fluorouracil + doxorubicin + cyclophosphamide (FAC) Cisplatin + doxorubicin	500 mg/m^2^ + 50 mg/m^2^ + 500 mg/m^2^ 70 mg/m^2^ + 55 mg/m^2^	3 3	1
Cisplatin + doxorubicin Docetaxel	60 mg/m^2^ + 60 mg/m^2^ 75 mg/m^2^	3 2	1
Cisplatin + doxorubicin	50 mg/m^2^ + 50 mg/m^2^	6	3
	75 mg/m^2^ + 50 mg/m^2^	5	1
	60 mg/m^2^ + 60 mg/m^2^	6	2
	60 mg/m^2^ + 60 mg/m^2^	4	1
	75 mg/m^2^ + 50 mg/m^2^	4	1
	50 mg/m^2^ + 60 mg/m^2^	6	1
Carboplatin + docetaxel	AUC-5 + 60 mg/m^2^	6	1

All 12 patients (100%) underwent therapeutic mastectomies on the affected side and risk reductive mastectomies on the contralateral side if applicable, except one patient who refused risk reductive mastectomy. A total of 23 mastectomies were performed in 12 patients: three simple, six skin-sparing and 14 nipple-sparing mastectomies. In 10 out of 12 cases immediate reconstruction was carried out. Five patients (42%) had prophylactic salpingoophorectomies performed after breast surgery. The mean observation time after breast surgery was 24 (range one - 63 months). Toxicity was not assessed in this evaluation.

## Results

Ten (83%) out of 12 patients achieved pCR after neoadjuvant chemotherapy. Two patients (17%) had residual cancer after NACT with partial response or stable disease. The proportions of the subgroups of those who achieved pCR are presented in Table 3. The pCR rates according to clinical stage I, II and III were 100%, 86% and 50%, respectively. Eight (80%) out of 10 patients with confirmed negative estrogen and progesterone receptor status achieved pCR. Five (83%) out of six patients with positive lymph node status prior to surgery achieved pCR. (Similarly, five (83%) out of six patients with node-negative disease achieved pCR).

**Table 3:** Proportions of patient subgroups achieving pCR

Characteristic	No. with pCR	Total no.	% PCR
Age, years			
20–40	8	8	100
41–50	1	2	50
51–60	1	2	50
Stage			
I	3	3	100
II	6	7	86
III	1	2	50
Estrogen receptor			
Positive	1	1	100
Negative	8	10	80
Missing	1	1	100
Lymph nodes			
Positive	5	6	83
Negative	5	6	83

Eleven patients are alive today, and one patient died due to brain metastases 15 months after breast cancer diagnosis. All 10 patients with pCR are disease free to date according to the available data. The mean observation time for the patient group with pCR is 25 months (range 6 – 63 months). Table 4 presents the characteristics of the two patients who did not achieve pCR.

**Table 4:** Characteristics of patients not achieving pCR

Patient	1	2
Age	49	56
cTNM/pyTNM	T2N0M0/pyT2N0M0	T3N3M0/pyT3N3aM0
Histology	Ductal carcinoma	Ductal carcinoma
Grade	3	3
ER/PR/HER2	Triple negative	Triple negative
NACT regimen	FAC (1000 mg + 100 mg + 1000 mg) 3 cyclesCisplatin+Doxorubicin (140 mg + 110 mg) 3 cycles	Cisplatin+Doxorubicin (80 mg + 90 mg 4 cycles, 80 mg + 85 mg 2 cycles)
Pre-tx tumor size	45 mm	X (extensive, diffuse, difficult to evaluate the size)
Post-tx tumor size	25 mm	65 mm
Operation	Unilateral mastectomy	Bilateral mastectomy
Lymphadenectomy	Yes	Yes
Adjuvant CT	No	No
Postoperative radiation	Yes	Yes
Progression	No	Brain metastases, PFS 12 months, OS 15 months

## Discussion

Our findings from the retrospective evaluation at the breast unit of PSCUH suggested that platinum-based neoadjuvant chemotherapy was highly effective in breast cancer patients with *BRCA1* gene mutations. In our patient group, 12 patients received platinum-based NACT. pCR was observed in10 patients (83%), and partial remission or stable disease was observed in two patients (17%). T. Byrski et al. [4] were the first to apply platinum-based chemotherapy in studies targeting *BRCA* mutation carriers specifically. As seen in Table 5, the pCR rates ranged from 59% to 83% in the evaluated studies. Our results matched these findings and confirmed the sensitivity of BRCA-associated breast cancer to platinum agents. In the neoadjuvant studies by T. Byrski [4, 5] and V. Moysienko [6], the authors applied cisplatin monotherapy. The patients in the current evaluation received cisplatin and doxorubicin (one patient received carboplatin and docetaxel). There were no available data on this combination for patients with BRCA-associated breast cancer. The results indicated that the addition of anthracycline to a platinum agent in the neoadjuvant setting produces equivalent or better results than monotherapy in terms of pCR. Most of the12 patients included in this evaluation had cancers that were diagnosed early. Seven patients had stage II, three patients had stage I, and two patients had stage III. These characteristics are comparable to the trial of T. Byrski et al. [5], where a pCR of 61% was achieved.

**Table 5:** Platinum-based neoadjuvant chemotherapy in BRCA-associated breast cancer

Author	n	Setting	Regimen	Efficacy
Byrski et al. 2010 [4]	12	Neoadjuvant	Cisplatin	pCR rate: 83%
Byrski et al. 2014 [5]	107	Neoadjuvant	Cisplatin	pCR rate: 61%
Moiseyenko et al. 2015 [6]	5	Neoadjuvant	Cisplatin	pCR rate: 60%
Sharma et al. 2016 [13]	30	Neoadjuvant	Carboplatin+Docetaxel	pCR rate: 59%

The BRCA1 founder mutations in our study included c.4035delA (BIC: 4154delA) and c.5266dupC (BIC: 5382insC). These are the same mutations examined in the study by Byrski et al.; however, C61G was also included in their study population but not in ours. Our results need to be verified in populations with other mutations.

The protocols for all patients in the reviewed neoadjuvant studies included the administration of the standard AC regimen as the adjuvant chemotherapy regardless of the level of response. The patients who achieved pCR in our study did not receive any adjuvant chemotherapy. Our observation times for patients with pCR varied from 1month to 63 months, and currently, the mean observation time is 25 months. All patients except one are still alive according to the available data. A comprehensive assessment of the survival rate of our platinum-based NACT will require additional follow-up. Observing the progression-free survival (PFS) rate of our patient group with pCR will provide highly interesting information in the future. No studies have properly assessed PFS that is specific to breast cancer patients with *BRCA1* mutations receiving only neoadjuvant platinum-based chemotherapy without adjuvant treatment.

Platinum agents also showed efficacy for *BRCA1* mutation carriers with advanced breast cancer. A *BRCA1* gene mutation was a good predictor of an increased response rate compared with wild-type BRCA1 [7–9]. The best evidence on platinum efficacy compared to standard therapy to date is presented by Telli et al. in the TNT trial [7]. The authors randomized carboplatin vs docetaxel in patients with metastatic TNBC. They observed a carboplatin response rate of 68% vs a docetaxel rate of 33% (*P* = 0.03) in the subgroup of 43 patients having *BRCA1/2* mutations. Further research is needed on metastatic BRCA-associated breast cancer.

There is a substantial amount of research on TNBC. In a study comprising 1824 patients with TNBC unselected for family history, 8.5% had a BRCA1 mutation, and 2.7% had a BRCA2 mutation [10]. The patients with BRCA-associated breast cancer represent a small subset of breast cancer patients. This is probably the reason for the lack of data for this specific patient population. To date, there are no available data from randomized trials that specifically target *BRCA1* gene mutation carriers with breast cancer. Evidence from the Maksimenko study [11] indicated that carriers of a germline *BRCA1* founder mutation (4153delA a 5382insC) had significantly improved prognoses at any stage compared to carriers of wild-type BRCA who had TNBC, even though only 7.8% of patients in this study had received platinum-based chemotherapy. These results may indicate that *BRCA1* mutation carriers have higher chemosensitivity.

The toxicity of chemotherapy was not evaluated. Platinum agents are known to cause mild to severe toxicity [12]. Cisplatin seems to be the drug that has the most evidence of efficacy in BRCA-associated breast cancer. No evidence from randomized trials exists on which platinum agent is more effective in BRCA-associated breast cancer. Further data are needed. A transition from cisplatin to carboplatin-based chemotherapy would likely benefit patients in terms of fewer side effects and higher tolerance to recommended platinum dosages.

## Conclusion

The results of the study confirm the high pCR rate of BRCA1-positive breast cancer after platinum-based neoadjuvant chemotherapy. Larger randomized studies and longer follow-up times are necessary to evaluate the role of platinum-based therapies in BRCA1-positive breast cancer.

## Abbreviations

NACT: Neoadjuvant chemotherapy
OS: Overall survival
pCR: Pathological complete remission
PFS: Progression-free survival
PSCUH: Pauls Stradins Clinical University Hospital
TNBC: triple negative breast cancer
